# Examination and Scientific Analysis of Thoracic Vertebral Fractures

**DOI:** 10.7759/cureus.44938

**Published:** 2023-09-09

**Authors:** Gurbinder Singh, Varun Rao, Aish Thamba, Dylan Roth, Mohamed A Zaazoue

**Affiliations:** 1 Department of Orthopaedic Surgery, University of California San Francisco, San Francisco, USA; 2 Department of Neurological Surgery, Indiana University School of Medicine, Indianapolis, USA

**Keywords:** preventative care, injury epidemiology, injury risk, vertebral fracture, thoracic vertebra

## Abstract

Background

Thoracic vertebral fractures are clinically important due to their association with the thoracic spinal cord and the potential to cause devastating neurological injury. Using the National Electronic Injury Surveillance System (NEISS) data, this study investigated fracture patterns to understand associated factors to improve prevention strategies. We explored different factors associated with thoracic vertebral fractures to improve our understanding of preventative strategies and patient care standards, focusing on spatial distribution, sex-age dynamics, and location of injury.

Methodology

This retrospective, cross-sectional study examines thoracic vertebral fractures across diverse age groups from 2013 to 2022, utilizing the NEISS database from the U.S. Consumer Product Safety Commission. Inclusion criteria based on specific terms related to thoracic fractures were employed. Descriptive statistics illustrated fracture distribution by age groups and associated products. Statistical analyses, including chi-square tests and multivariate logistic regressions, were conducted to explore associations between fracture occurrence, locations, products, age, and gender.

Results

The analysis of thoracic vertebral fractures by location and associated products yielded several statistically significant findings. Notably, the prevalence of fractures at home (39.67%) was significantly higher than in other locations, and these differences in fracture distribution were statistically significant (χ² = 7.34, p < 0.001). Among the associated products, ladders (10.46%) emerged as the most frequent product associated with fractures. Multivariate logistic regression analysis showed that the age groups of 41-50, 51-60, and 61-70 had increased odds of fractures with adjusted odds ratios (AORs) of 1.08 (95% confidence interval (CI) = 1.04-1.42, p < 0.05), 1.21 (95% CI = 1.13-1.56, p < 0.001), and 1.17 (95% CI = 1.08-1.39, p < 0.001), respectively. The likelihood of thoracic vertebral fractures did not significantly differ between males and females (AOR = 1.12, 95% CI = 0.87-1.53, p = 0.262). Fracture distribution by age groups and products indicated increasing ladder-related fractures within the 41-50 age group and 51-60 age group. Football-related fractures peaked within the 21-30 age group. Fracture distribution patterns for bicycles had increased prevalence within the 11-20 and 21-30 age groups, and football-related fractures in younger age groups.

Conclusions

This study analyzed factors associated with thoracic vertebral fractures, showing the significance of targeted preventative interventions, such as earlier screening, physical therapy, and nutritional status assessment, in the setting of significant location and age-related susceptibilities. The observed patterns of injury provide a foundation for future research to elucidate the underlying mechanisms between different environments and the likelihood of injury to improve preventive strategies.

## Introduction

Thoracic vertebral fractures are a subject of heightened medical significance due to their potential risk of causing devastating neurological injuries. Researchers and clinicians alike have worked on studying the intricate interplay with location, demographic factors such as sex and age, and the utilization of different consumer products or activities [1,2]. These fractures, impacting the vertebral bodies within the thoracic spine, carry significant implications for patient well-being, encompassing varying degrees of morbidity, mortality, and enduring consequences [3]. An in-depth exploration of the multifaceted landscape surrounding thoracic vertebral fractures is indispensable for shaping effective treatment paradigms and elevating the standards of patient care.

The location factor takes center stage as a pivotal determinant of fracture characteristics, unveiling the nuanced spatial distribution of injuries within the thoracic vertebral column. The intricate anatomical architecture of the spine imparts distinct biomechanical profiles to different regions, influencing fracture patterns and severity [4]. A comprehensive understanding of this distribution facilitates tailored interventions and prognostication of potential complications.

The dynamics of sex and age introduce further complexity to the epidemiology of thoracic vertebral fractures. Emerging research suggests potential sex-specific predilections for specific spinal fractures, possibly rooted in divergent bone density and hormonal influences. Meanwhile, age, a formidable predictor of fracture incidence, exerts its effect through dynamic alterations in bone quality and strength [5,6]. Exploring sex and age dynamics within the context of thoracic vertebral fractures is pivotal for revealing underlying risk factors and optimizing strategies for fracture prevention [1,7,8].

We hypothesize that fractures exhibit distinct distribution patterns across various locations, influenced by discernible age- and sex-related dynamics. Furthermore, we emphasize the significant role of medical products in shaping strategies for fracture management. Through an analysis of data sourced from the National Electronic Injury Surveillance System (NEISS) database, this study aims to evaluate our hypothesis, seeking to illuminate the epidemiology and pathophysiology of thoracic vertebral fractures. Our ultimate goal is to establish the groundwork for more refined clinical interventions and preventative measures aimed at enhancing patient outcomes.

## Materials and methods

This study adopts a cross-sectional research design to investigate the occurrence of thoracic vertebral fractures within diverse age groups over 10 years from 2013 to 2022. The principal data source for this analysis is the NEISS database, overseen by the U.S. Consumer Product Safety Commission (CPSC) and utilized in multiple prior analyses [7-15]. This extensively curated database aggregates information from approximately 100 U.S. hospitals, meticulously selected as a probability-based sample from a pool of 6,100 hospitals, each equipped with a minimum of six beds and a 24-hour emergency department. This dataset’s de-identified data encompass unintentional consumer-related injuries and fatalities tied to consumer products, encapsulating variables such as implicated products, injured body parts, age, gender, and locations of injury incidents. The data is anonymized, cannot be linked to specific individuals, and is exempted from Institutional Review Board approval as it qualifies in the exempt human subject research category.

The study participants encompass individuals who experienced thoracic vertebral fractures within the stipulated 10-year timeframe. The process commenced with a systematic effort to isolate and classify lower trunk injuries as fractures. Each injury narrative underwent careful evaluation against well-defined inclusion and exclusion criteria. Inclusion criteria were entries with terms such as “Thoracic,” “Thorac,” “T-spine,” and “T-spinal,” in conjunction with designations “T1,” “T2,” “T3,” “T4,” “T5,” “T6,” “T7,” “T8,” “T9,” “T10,” “T11,” and “T12.” With adaptation from prior literature, these chosen terms encompassed fractures directly pertinent to the thoracic spine. Study participants were distributed across nine distinct age groups (in years) as follows: “0-10,” “11-20,” “21-30,” “31-40,” “41-50,” “51-60,” “61-70,” “71-80,” and “81 and above.” This ensured alignment with the established age stratification protocols from previous research methodologies [1,2,4,5,16].

Descriptive statistics were utilized to present the distribution of thoracic vertebral fractures across various age groups and their associations with specific products. Statistical analysis was conducted to explore the impact of implicated products, injured body parts, age, gender, and locations of injury incidents. The analysis focused on the top five locations for thoracic vertebral fractures based on previous studies, while excluding unknown locations due to their lack of substantial public health significance [6,17]. Pearson chi-square tests were employed to assess potential significant differences in thoracic vertebral fracture occurrence across different areas (“Home,” “Place of Recreation/Sports,” “Other Public Property,” “Street or Highway,” “School”) and products (“Ladder,” “Floor,” “Bicycles,” “Football,” “Bed”). Per previous investigations [1,6,17], multivariate logistic regression was utilized to assess patterns of both sex and age groups. This involved adjusting for age as a covariate in the regression model examining the relationship with sex. Similarly, changing sex as a covariate in the regression model examines the relationship with age. Through these analyses, calculations were undertaken to derive adjusted odds ratios (AOR) in conjunction with their corresponding 95% confidence intervals (CI) and accompanying p-values. The reference category for the age group was set as 10-20 years and male sex. An AOR >1 indicated an increased likelihood of a spine injury being linked to the female sex and certain age groups compared to the reference group (males and other age groups). Conversely, AOR values <1 signaled a decreased probability of females and specific age groups experiencing a spine injury relative to their male counterparts and other age groups.

## Results

A total of 398,261 thoracic vertebral fractures were included in this study. The different locations and associated products for these fractures are presented in Table 1. The distribution of fractures varied significantly across other locations. The highest incidence was observed in the home setting, constituting 39.67% of the total cases. Fractures occurring at places of recreational or sports activities accounted for 17.81% of the cases, whereas fractures on other public properties, streets or highways, and schools represented 6.71%, 5.54%, and 3.41% of the total fractures, respectively. The distribution of fractures across these locations exhibited statistical significance (χ² = 7.34, p < 0.001).

**Table 1:** Distribution of thoracic vertebral fractures by location and associated products. Distribution of thoracic vertebral fractures by location and associated products, with Pearson χ² = 7.34, p < 0.001, indicating significant variation across different locations and products.

	Number of fractures (%)	χ^2^, P-value
Location		
Home	158,003 (39.67)	χ^2^ = 7.34, p <0.001
Place of recreation/Sports	70,919 (17.81)	
Other public property	26,736 (6.71)	
Street or highway	22,063 (5.54)	
School	13,583 (3.41)	
Product associated with fracture		
Ladder	41,657 (10.46)	χ^2^ = 2.94, p = 0.861
Floor	33,713 (8.47)	
Bicycles	22,235 (5.58)	
Football	19,021 (4.78)	
Bed	18,413 (4.62)	

Regarding products associated with the fractures, most cases involved ladders (10.46%), followed by floor-related incidents (8.47%). Bicycles, football, and beds contributed to 5.58%, 4.78%, and 4.62% of the fractures, respectively. No statistically significant association was observed between the type of product associated with fractures and the incidence of fractures (χ^2^ = 2.94, p = 0.861).

Table 2 displays the logistic regression analysis results investigating the relationship between thoracic vertebral fractures and sex and age.

**Table 2:** Logistic regression of thoracic vertebral fractures by sex and age. Presentation of the outcomes derived from the logistic regression analysis revealing patterns in thoracic vertebral fractures stratified by sex and age groups, featuring adjusted odds ratios and associated confidence intervals. Age groups “Age^a^” are adjusted for sex, while sex categories “Sex^b^” are adjusted for age.

	N (%)	AOR (95% CI)	P-value	
Age^a^				
0–10	13,082 (3.28)	0.98 (0.48–1.51)	0.876	
11–20	16,483 (4.14)	[Ref]	[Ref]	
21–30	33,090 (8.31)	1.32 (0.73–1.65)	0.668	
31–40	49,047 (12.32)	1.01 (0.35–1.19)	0.084	
41–50	63,886 (16.04)	1.08 (1.04–1.42)	<0.05	
51–60	77,413 (19.44)	1.21 (1.13–1.56)	<0.001	
61–70	70,403 (17.68)	1.17 (1.08–1.39)	<0.001	
71–80	55,975 (14.05)	1.02 (0.91–1.46)	0.383	
81 and above	19,814 (4.98)	0.83 (0.52–1.51)	0.407	
Sex^b^				
Male	197,601 (49.62)	[Ref]	[Ref]	
Female	200,660 (50.38)	1.12 (0.87–1.53)	0.262	

The multivariate logistic regression analysis revealed that age groups of 11-20, 21-30, 31-40, 41-50, 51-60, 61-70, and 71-80 displayed varying associations with thoracic vertebral fractures compared to the reference age group (11-20 years). Notably, individuals within the age group of 41-50 exhibited a statistically significant increase in the odds of thoracic vertebral fractures 1.08 (95% CI = 1.04-1.42, p < 0.05). Similarly, individuals aged 51-60 and 61-70 had significantly elevated odds of thoracic vertebral fractures with AORs of 1.21 (95% CI = 1.13-1.56, p < 0.001) and 1.17 (95% CI = 1.08-1.39, p < 0.001), respectively.

Regarding sex, the logistic regression analysis indicated that the likelihood of thoracic vertebral fractures did not significantly differ between males and females 1.12 (95% CI = 0.87-1.53, p = 0.262).

**Figure 1:**
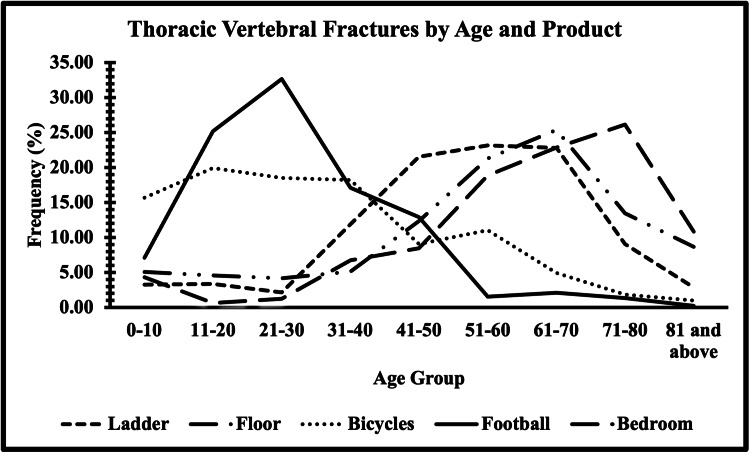
Thoracic vertebral fractures by age and product. This figure depicts the distribution of fractures across age groups and associated products derived from the data presented in Table 3, illustrating variations in prevalence percentages for different age ranges.

Table 3 provides a comprehensive overview of the distribution of thoracic vertebral fractures across different age groups and their association with specific products. The data reveals varying patterns of fracture occurrence across age groups and products. Noteworthy trends include the increasing prevalence of fractures related to ladders with advancing age, particularly within the 41-50 age group (21.54%) and 51-60 age group (23.17%). Conversely, bicycle-related fractures were more prevalent among younger age groups, such as the 11-20 age group (19.92%) and the 21-30 age group (18.50%). Football-related fractures displayed a distinctive pattern, peaking within the 21-30 age group (32.64%) (Figure 1). Furthermore, the distribution of fractures associated with beds consistently increased with age, reaching its highest proportion within the 71-80 age group (26.14%) (Figure 1).

**Table 3:** Distribution of fractures by age groups and associated products. Display of fracture distribution across distinct age groups, revealing varying prevalence percentages and showcasing correlations between fractures and specific products.

	0–10	11–20	21–30	31–40	41–50	51–60	61–70	71–80	81 and above
Ladder	3.27	3.35	2.13	11.90	21.54	23.17	22.82	9.05	2.84
Floor	5.08	4.57	4.16	5.13	12.34	21.28	25.40	13.45	8.66
Bicycles	15.67	19.92	18.50	18.22	9.06	11.06	4.91	1.83	0.99
Football	7.11	25.19	32.64	17.10	12.89	1.55	2.08	1.34	0.24
Bedroom	4.32	0.61	1.22	6.76	8.44	18.85	22.84	26.14	10.83

## Discussion

Our study findings focus on the associations between location, demographic factors, products, and activities that led to thoracic vertebral fractures. Most fractures (39.67%) occurred at home, while recreational facilities contributed to 17.81% of cases. Fractures in other public settings and schools represented smaller percentages. The contrast of the distribution of thoracic vertebral fractures according to location underscores the significance of understanding the contextual stages where such injuries occur. Notably, the home setting emerged as the locus of the highest proportion of fractures, a finding aligned with previous research that underscores the significance of preventive measures within domestic environments (Table 1). Fractures associated with recreational and sports activities, public properties, streets, highways, and schools each highlighted distinct contexts of risk exposure. These findings emphasize the need for targeted interventions tailored to these specific environments, enhancing injury prevention strategies. However, there needs to be additional research conducted into the influence of environmental safety on the likelihood of thoracic vertebral injury as ecological safety is an integral component of the holistic evaluation and management of vertebral fractures and the health of at-risk populations.

Specific targeted interventions exist in managing thoracic vertebral fractures, especially in at-risk populations more vulnerable to experiencing fragility fractures due to decreased bone mineral density and/or falls. Clinicians must be aware of available assessment tools and management strategies to identify and prevent vertebral fractures, such as imaging modalities, fracture risk assessment to estimate 10-year fracture risk, bone densitometry through the dual-energy X-ray absorptiometry scan, nutritional status assessment, and physical and occupational therapy measures to enhance musculoskeletal agility and pharmacological interventions. Subsequently, the literature demonstrates the utilization of fracture liaison services to improve the identification and resource utilization of available therapies to reduce the likelihood of fractures in patient populations at risk for vertebral fractures [1,18-20].

Another pertinent finding is that ladders were the most common product associated with fractures (10.46%), followed by floor-related incidents (8.47%). Logistic regression analysis explored the impact of age and sex on fractures, revealing significant associations for specific age groups (41-50, 51-60, and 61-70) but no significant sex-based difference. The distribution of fractures by age groups and products highlighted distinct patterns such as ladder-related fractures increasing with age, bicycle-related fractures being more prevalent among younger age groups, football-related fractures peaking in the 21-30 age group, and bed-related fractures rising with age and being more frequent in the elderly [21,22].

Of note, the influence of sex and age on thoracic vertebral fractures emerges as a compelling facet of our study (Table 2). While the study period exhibited a pronounced association with fracture likelihood, sex did not appear to influence fracture incidence significantly. The rise in fracture odds with advancing age underscores the dynamic interplay between skeletal health and age-related changes. The increased likelihood of fractures in the 41-50 age group indicates a biomechanical vulnerability during this period. However, as individuals age, thoracic spine fractures increase for both men and women due to age-related bone density loss, degenerative changes in the spine, and the increased risk of falls. Due to the interplay between osteoporosis and sarcopenia, literature has shown an increased risk of fragility fractures in vertebrae with aging. Fragility fractures reduce the quality of life of older adults through significant psychosocial detriment due to loss of autonomy and increased likelihood of progression toward disability and mortality [18,23-25].

Furthermore, postmenopausal women are at higher risk of fractures due to the accelerated decline in bone density associated with hormonal changes. This study finding necessitates further exploration to elucidate the underlying mechanisms contributing to this phenomenon. The absence of a substantial sex-based effect underscores the complex interplay of factors governing fracture risk, emphasizing the need for more nuanced investigations. A probable postulation for this finding could be attributed to the increased frequency of injuries in postmenopausal females is balanced by injuries in similar-aged males who are using ladders or participating in do-it-yourself home projects more frequently than females. The predictive value of locations and objectives that can be attributed to influencing the risk of thoracic vertebral fractures in these populations is uncertain. However, additional research could potentially aid in reducing the burden of vertebral fractures in a rapidly aging population [1,23,26-28].

The presence of specific items in various environments as contributing factors to thoracic vertebral fractures merits careful consideration (Table 1). Ladders, floor-related incidents, bicycles, football, and beds emerged as noteworthy contributors. Despite the absence of a statistically significant association between the type of product and fracture incidence, the distribution of fractures across products provides insights into distinct risk scenarios. Further exploration into the circumstances leading to these associations could yield valuable insights for preventive strategies and public awareness campaigns. The available data suggests that in terms of absolute numbers, men tend to have a slightly higher incidence of thoracic spine fractures than women, particularly in younger age groups. This is often attributed to higher rates of trauma-related incidents and participation in activities with a higher risk of injury among younger men [2,3,5,6,20-22,27].

Our study has several significant limitations to acknowledge. While the NEISS database offers a comprehensive dataset, its reliance on reported cases may introduce underreporting or selection bias. Moreover, the dataset’s focus on acute injuries limits our understanding of long-term consequences and subsequent health impacts. The presence of approximately 30% of thoracic vertebral fractures with unknown locations restricts the study’s scope to analyzing only those fractures with documented locations, potentially limiting the comprehensiveness of the findings. Furthermore, some fractures may be managed in a subacute or chronic setting and may, therefore, not present in emergency departments and instead be managed in outpatient settings. Future studies could consider integrating longitudinal data to explore the trajectory of thoracic vertebral fractures and their implications over time.

The implications of our findings resonate with clinical practice, public health initiatives, and injury prevention strategies. The identification of risk contexts and age-related vulnerabilities informs targeted interventions. Moreover, the interplay of comorbid conditions such as osteoporosis in a rapidly aging population and vertebral fractures highlights the need for holistic assessment and management to prevent vertebral fractures and the importance of patient education [2,18,20,24,27].

Given these considerations, this study underscores several directions for future research (Table 3). Investigating the biomechanical intricacies underlying age-related vulnerabilities and discerning the mechanisms driving sex-related fracture differences would contribute substantially to our understanding. Longitudinal studies exploring the transition from acute injury to chronic conditions are warranted to determine the trajectory of recovery and the implications for quality of life. Furthermore, detailed analyses of product-related fractures could inform product design, regulations, and guidelines for consumer safety.

## Conclusions

This comprehensive study on thoracic vertebral fractures has illuminated the intricate interplay of factors that shape their epidemiology and pathophysiology. The findings have highlighted the pivotal role of location, age, and certain products or activities in influencing the occurrence and characteristics of these fractures. The home environment emerged as a significant locus of fractures, emphasizing the need for targeted preventive measures in domestic settings. Age-related vulnerabilities, especially in the 41-70 age groups, underscore the dynamic relationship between skeletal health and aging, with implications for fracture risk. While sex did not appear to be a significant predictor of fracture incidence, the study emphasized the multifaceted nature of fracture risk factors. Importantly, the presence of specific items in various environments as contributors to fractures warrants further investigation for potential preventive strategies. Despite limitations, this study serves as a valuable foundation for future research directions, including biomechanical intricacies, longitudinal studies, and product-related fracture analyses. These insights have far-reaching implications for clinical practice, public health initiatives, and injury prevention strategies aimed at improving patient outcomes and reducing the burden of thoracic vertebral fractures, particularly in an aging population.
